# Haste or Speed? Alterations in the Impact of Incentive Cues on Task Performance in Remitted and Depressed Patients With Bipolar Disorder

**DOI:** 10.3389/fpsyt.2018.00396

**Published:** 2018-09-03

**Authors:** Henry W. Chase, Jay C. Fournier, Haris Aslam, Richelle Stiffler, Jorge R. Almeida, Barbara J. Sahakian, Mary L. Phillips

**Affiliations:** ^1^Department of Psychiatry, University of Pittsburgh School of Medicine, Pittsburgh, PA, United States; ^2^Department of Psychiatry, Dell Medical School, University of Texas at Austin, Austin, TX, United States; ^3^Department of Psychiatry, University of Cambridge, Cambridge, United Kingdom

**Keywords:** bipolar disorder, reinforcement (psychology), reaction time, motivation, major depressive disorder

## Abstract

A variety of evidence suggests that bipolar disorder is associated with disruptions of reward related processes, although the properties, and scope of these changes are not well understood. In the present study, we aimed to address this question by examining performance of patients with bipolar disorder (30 depressed bipolar; 35 euthymic bipolar) on a motivated choice reaction time task. We compared performance with a group of healthy control individuals (*n* = 44) and a group of patients with unipolar depression (*n* = 41), who were matched on several demographic variables. The task consists of an “odd-one-out” discrimination, in the presence of a cue signaling the probability of reward on a given trial (10, 50, or 90%) given a sufficiently fast response. All groups showed similar reaction time (RT) performance, and similar shortening of RT following the presentation of a reward predictive cue. However, compared to healthy individuals, the euthymic bipolar group showed a relative increase in commission errors during the high reward compared to low condition. Further correlational analysis revealed that in the healthy control and unipolar depression groups, participants tended either to shorten RTs for the high rather than low reward cue a relatively large amount with an increase in error rate, or to shorten RTs to a lesser extent but without increasing errors to the same degree. By contrast, reward-related speeding and reward-related increase in errors were less well coupled in the bipolar groups, significantly so in the BPD group. These findings suggest that although RT performance on the present task is relatively well matched, there may be a specific failure of individuals with bipolar disorder to calibrate RT speed and accuracy in a strategic way in the presence of reward-related stimuli.

## Introduction

Bipolar disorder (BD) is characterized by modified behavioral and neural responsiveness to reward (1–3). Although individuals with BD report a heightened desire to attain happiness (4), patients' lives are typically characterized by instability and low levels of (eudaimonic) well-being (5). One potential explanation for this discrepancy is that there is an imbalance in the neural systems controlling the pursuit of reward. Responsiveness to reward in BD may better reflect the arousing or activating properties of reinforcement-related stimuli rather than enhanced hedonic responses (6), akin to a non-specific invigoration of behavior elicited by reward-related stimuli (7). In general, invigoration is likely to be adaptive in a reward-rich environment, but there may also be deleterious consequences [e.g., (8)] which may be relevant for BD.

Alterations in the performance of BD on reinforcement learning paradigms have been reported (9, 10), as well as altered decision making (11), but these studies vary in their emphasis on risk taking, blunted reward sensitivity and cognitive flexibility. In the present study, we employed the Cued Reinforcement Reaction Time (CRRT) task, a motivated reaction time task which has been employed in studies of 5-HT (serotonin) manipulations (12) and patients (13, 14). The reinforcement contingencies on the task should prompt the participant to shorten their reaction times on high reinforcement trials compared to the low trials, so primarily the task is used as an index of the motivational impact of cues on a simple cognitive task. In a previous investigation with the CRRT (12), manipulation of central nervous system 5-HT using acute tryptophan depletion (ATD) reduced reinforcement related speeding in a group of healthy individuals. In addition, the pattern of errors changed, with a relative reduction of errors in the high reinforcement condition.

With regard to BD, we considered two hypotheses. First, there may be generally inflated urgency across all rewarding contexts, or a preference for speed over accuracy. Second, there may be an impairment in the contextual calibration of urgency. It is often assumed that increasing impulsivity is broadly maladaptive [e.g., (15)]. However, there is also a concept of “functional” impulsivity (16), in which rapid if inaccurate decisions are necessary in order to obtain reward and are thereby adaptive. Thus, impaired calibration could lead to hasty and inaccurate decision making in reward-sparse environments when accuracy is needed, but also excessively conservative decision making in reward-rich environments. We performed a detailed analysis of task performance to investigate these possibilities.

We recruited four groups of participants (bipolar euthymic, bipolar depressed, major depressive disorder (MDD), and healthy controls), and hypothesized that the normal pattern of reward-related speeding would be altered in individuals with bipolar disorder. Note that these predictions are focused on the notion that BD patients (euthymic or depressed) would show altered impulsivity that would be detected on the CRRT paradigm. This design allows the impact of mood state to be largely differentiated from a BD-related trait abnormality. In a previous study with unipolar depressed patients (14), we observed an overall intact pattern of responding on the paradigm (i.e., similar reward-related speeding to controls), and even enhanced performance on some metrics. We also sought to determine whether a similar finding could be identified in the present data set. Our analytical strategy incorporated conventional reaction time (RT) and error difference scores. To provide further support to our conclusions, we supplemented this analysis with a simple reinforcement learning-based approach, instantiated within a general linear model (GLM).

## Methods

### Participants

Thirty currently depressed adults with bipolar disorder (BPD) type I, 35 currently Euthymic individuals with bipolar disorder (BPE) type I, and 41 currently depressed adults with MDD participated in the study. All BPE/BPD/MDD participants were diagnosed according to DSM-IV criteria using the Structured Clinical Interview for DSM-IV-Research-Version [SCID-P: (17)]. All BPD and MDD participants were in a Major Depressive Episode, as determined by SCID-P criteria, at the time of a functional magnetic resonance imaging (fMRI) scan which occurred ~1 week prior to the behavioral testing session at which the present data was collected. Data obtained from this scanning session have been previously reported (6, 18–20). Current mood state was confirmed by having a Hamilton Rating Scale for Depression (HRSD-25) ≥17 (21) and a Young Mania Rating Scale [YMRS: (22)] score ≤10 on the day of the test (unless it occurred < 4 days after the scanning session), although three BPD and five MDD individuals meeting SCID-P criteria for depressive episode had a HRSD-25 score between 11 and 17 on the test day, while three BPE had scores of 17 or over. All participants also completed the Spielberger State Anxiety Inventory on the test day (23). Prevalence of lifetime comorbid anxiety and substance use disorders are reported in Table 1. Importantly, all BPE/BPD/MDD participants were free from alcohol/substance abuse or dependence for a minimum of 3 months prior to the study (range: 4–235 months). Forty-four healthy adult control participants (HC) with no previous personal or family history of psychiatric illness in first-degree relatives participated in the study. All HC participants were also free of previous or current alcohol/illicit substance abuse. All participants were right-handed and native English speaking. The study protocol was approved by the University of Pittsburgh Institutional Review Board. After complete description of the study to the participants, written informed consent was obtained.

**Table 1:** Demographic and clinical information for participants in the four groups.

	**Healthy controls**	**Major depressive disorder**	**Bipolar depression**	**Bipolar euthymic**	**Group differences**
Gender (M:F)	15:29	9:32	6:24	12:23	*X*^2^ = 3.19, *p* = 0.36
Age (years; mean/S.D.)	33.60 (6.093)	32.29 (7.74)	32.15 (8.85)	32.62 (8.08)	*F*_(3, 146)_ < 1
NART IQ (mean/S.D.)	112.32 (9.53)	112.32 (9.53)	110.22 (10.047)	112.97 (9.30)	*F*_(3, 146)_ < 1
Years of Education (mean/S.D.)	6.41 (1.28)	6.10 (1.28)	5.30 (1.12)	6.31 (1.21)	*F*_(3, 146)_ = 5.47, *p* = 0.001 (BPD < all other groups)
HRSD 25 (mean/S.D.)	1.75 (1.97)	25.90 (6.14)	25.90 (6.099)	7.43 (6.87)	*F*_(3, 146)_ = 202.34, *p* < 0.001
Illness Duration (mean/S.D.)	N/A	13.95 (7.66)	16.41 (8.18)	13.074 (7.60)	*F*_(2, 103)_ = 1.57, *p* = 0.21
Number of Manic Episodes	N/A	N/A	1.80 (0.96)	2.23 (1.46)	*F*_(1, 63)_ = 1.89, *p* = 0.17
Number of Depressive Episodes	N/A	2.93 (1.37)	3.03 (1.33)	2.31 (1.66)	*F*_(2, 103)_ = 2.84, *p* = 0.063
YMRS (mean/S.D.)	0.41 (0.84)	3.17 (2.50)	4.33 (2.31)	2.31 (2.58)	*F*_(3, 146)_ = 24.71, *p* < 0.001
State Anxiety (mean/S.D.)	27.25 (7.85)	55.63 (10.087)	53.80 (11.31)	33.97 (10.92)	*F*_(3, 146)_ = 79.29, *p* < 0.001
Lifetime Comorbid Anxiety Disorders (W:WO)	0:44	30:11	22:8	11:24	
Lifetime Substance Use Disorders (W:WO)	0:44	15:26	12:18	14:21	
Psychotropic Medication Load (mean/S.D.)	0 (0)	2.44 (2.062)	3.53 (2.30)	3.09 (1.72)	*F*_(3, 146)_ = 33.77, *p* < 0.001 (HC < all patient groups, otherwise no significant differences)
Antipsychotic (T:NT)	0:44	3:38	19:11	22:13	
Antidepressant (T:NT) (includes Buproprion)	0:44	30:11	12:18	12:23	
Mood Stabilizer (T:NT)	0:44	6:35	16:14	23:12	
Anxiolytic (predominantly Benzodiazepine) (T:NT)	0:44	10:31	8:22	4:31	

*T:NT, taking: not taking; W:WO, with:without symptom*.

Further exclusion criteria for all participants included history of head injury, systemic medical illness, cognitive impairment, premorbid IQ estimate < 85 (as derived from the National Adult Reading Test: NART), schizophrenia/schizo-affective disorder and rapid cycling disorder. All included participants showed <21 errors per condition/block, and < 8 time-outs. The final participant numbers per group listed above and in Table 1 do not include a further three individuals with BPE and two with MDD who were excluded due to poor task performance (>9 time outs). A high number of timeouts might have been reflective of a failure to understand the instructions fully, and would have resulted in a reduction in the number of trials in which performance feedback was provided. Timeouts also reduce the amount of data available for modeling within the GLM. Two further participants (one BPE and one HC) were excluded on the basis of a relatively poor GLM fit (log likelihood <1100/Z score of the residual variance relative to overall mean Z>7; all included participants log likelihood = 1150–1350), which was generally reflective of abnormal performance (outliers) on one or more metric.

### Procedure

The paradigm was the same as that employed by Cools et al. (12). Participants performed two short practice blocks (20 trials) of a task, based on the circles task of Duncan et al. (24), in which they had to select the odd-one-out of three stimuli, within 2,000 ms. The mean and standard deviation of the participant's reaction time on the second practice block were recorded, and used as the “reward threshold” for the next stage of the task. Participants then performed two longer blocks (96 trials each) of a similar task, which contained a reinforcement component via the potential to win points. Points rewards were available on some trials, and were dependent on subjects' accuracy and speed, as well as the presence of rectangular cues which surrounded the area where the circles were presented, and were presented before the circles were presented. One cue predicted reward availability on 90% of trials (high probability cue), the second on 50% (medium probability cue), and the third on 10% of trials (low probability cue). Colors used for these cues were always red, blue and yellow, and subjects were randomly assigned to one of two mappings between cue and reward probability. Of the 96 trials in each block, 32 trials were performed with each cue type. There were 12 different arrays of three circles, and these were counterbalanced within cues such that there were no cue repetitions, and only two response repetitions occurred. If rewards were available on a given trial and the subject responded both correctly and faster than their reward threshold, the subject would receive 100 points, a green smiling face and a flourish sound. If the same were true, but the subject had responded slower than their reward threshold, they would receive 1 point, a green smiling face and a high frequency tone. Finally, if the subject was incorrect on trials where reward was available, they would receive negative feedback (a red frowning face and a low frequency tone, but no loss of points). If reward was not available on a given trial, no feedback was provided.

### Data analysis: basic analyses

First, raw RT data were transformed using a reciprocal transform [see (25)]. For basic analyses, mean reciprocal RTs from correct trials were calculated for each cue (high/medium/low) and block (first/second) separately. Repeated-measures ANOVA was then conducted for the resulting variables, including effects of block and cue as a within-participant factors, and the effect of group as a between-participant factor. Following previous studies (26), we also computed a measure of RT speeding, contrasting the high vs. low reward probability cue following Z transformation of the raw RT values (with reference to the mean and standard deviation for all cues within a block). We focused on block 2 only, as the reward contingencies were more likely to have been learned by that stage. All parametric analyses were confirmed by including years of education, age and gender as covariates. In all cases, findings were similar and these analyses are not reported. Following the suggestion of a reviewer, we also performed an additional analysis involving number of manic episodes in the bipolar groups alone (BPE/BPD).

Due to their non-normal distribution, error scores (per block/cue) were analyzed using non-parametric tests (Kruskal–Wallis test). Similar difference scores reflecting the effect of high vs. low probability cues for block 2 were calculated to compare with RT speeding metrics described above. Speed/accuracy relationships were investigated in two ways: first, with a simple Spearman's Rho correlation; second, using ordinal logistic regression. For the latter analysis, six error categories (−3 to +2 errors for high vs. low cue trials, block 2) containing roughly similar numbers of individuals were created to reduce model complexity.

### Data analysis: general linear model

As a follow up analysis, reciprocal RTs from the whole task (both blocks) were fit within a single general linear model [GLM: (25, 27)] separately for each participant. The goal of this modeling was broadly to demonstrate that the data are consistent with a Reinforcement Learning (RL) model, and that similar findings could be obtained using different modeling approaches. Our initial analyses suggested that the data did not provide strong constraints over multiple possible free parameters—specifically, the design did not allow unique identification of these parameters. We therefore set them either on the basis of prior work or to reflect a nominal magnitude ranking. These arbitrary decisions were justified insofar as they were unlikely to have a substantial impact on the overall pattern of data—at least with respect to group differences. To correct for autocorrelated properties of the timeseries, an AR(3) ARIMA model [see (25)] was fit using MATLAB (regARIMA function), and fixed effects at the subject level were thereby obtained. The model contained the following components as independent (predictor) variables: the first 5 trials per block [see (25)]; error trials; post error trials; a linear trend to correct for non-specific improvement on the task (trial number); and reward expectancy generated from a reinforcement learning model. The latter component was most relevant to our focus, and was derived from the following equation:

$$\begin{array}{l} \text{Q}\left(\text{cue},\text{t}\right)\leftarrow \text{Q}\left(\text{cue},\text{t}\right)+\text{alpha}*\left(outcome\left(t\right)-Q\left(cue,t\right)\right) \end{array}$$

The learning rate (alpha) was set to 0.2 for win or no-win outcomes [see (28)], but for punishment trials (signaled errors) it was set to 0.3 to reflect an increased salience of this condition. For 100 point wins, the outcome value was set to 3, and for 1 point wins it was set to 1. Trials on which responses were too slow were excluded from the analysis. We focused on the beta parameters associated with the Q-value regressor for further analysis, examining whether there were group differences using a combination of one-way ANOVA and a Bayesian test of the null hypothesis (29), and also recapitulated the ordinal logistic regression analysis described above using the *Q*-value beta value instead of the RT speeding measure.

### Effect of medication

We computed: (1) medication load, an index that reflects the number and dose of different medications, as in our previous neuroimaging studies on bipolar disorder [e.g., (30) and 2] identified medication status (taking vs. not taking each of five main psychotropic medication subclasses: mood stabilizers/antipsychotics/antidepressant/anxiolytics/dopaminergic-antidepressants, e.g., Bupropion: see Table 1). Medication load calculation was based on a binary categorization of low and high dose groupings of antidepressants and mood stabilizers; a binary categorization of antipsychotics relative to mean effective daily dose of chlorpromazine hydrochloride; and a binary categorization of benzodiazepine dose relative to the recommended daily dose of each type of benzodiazepines. If a participant was not taking a given class of the medication, they would receive a score of 0, a low dose would receive a score of 1 and a high dose would receive a score of 2. Scores for each class of medication were added together to produce the final medication load score. Details of the patients' medications are presented in Supplementary Table 1. We tested whether the speed/accuracy relationships with the bipolar groups described within the ordinal logistic regression model were significant when medication (both main effect and medication by RT speeding interaction) was concurrently modeled.

## Results

### Demographic variables

The four groups were well matched for age, gender, and NART-estimated IQ (see Table 1). The groups differed on years of education, due to the BPD group showing a lower number of years of education.

### Reaction times and overall performance

**Figure 1:**
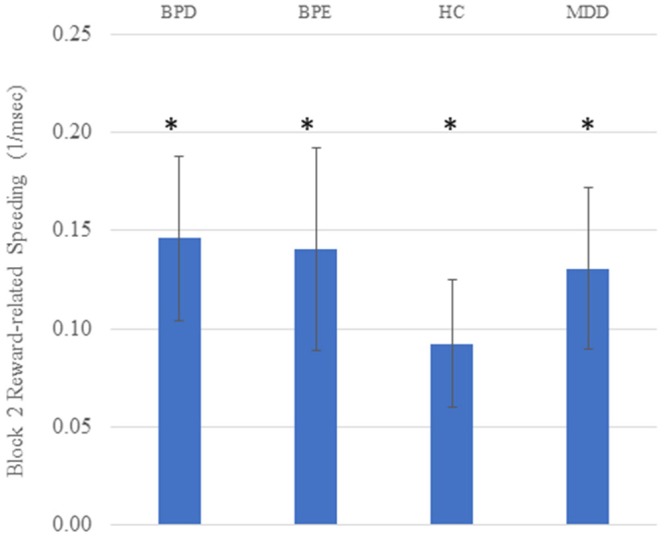
Reward-related speeding (high vs. low reward cue) on the second block in all four groups. Higher scores on the Y axis indicate faster responding for high vs. low reward cues. Units are 1/ms; error bars reflect standard error of the mean; asterisks reflect the mean value being significantly greater than zero (*p*'s < 0.05).

Analysis of variance (ANOVA) conducted on the reciprocal RTs revealed a significant main effect of block [*F*_(1, 146)_ = 48.38, *p* < 0.001], a significant main effect of cue [*F*_(2.00, 291.22)_ = 10.94, *p* < 0.001] and a significant block by cue interaction [*F*_(1.99, 290.98)_ = 15.73, *p* < 0.001]. The main effect of group was not significant [*F*_(3, 146)_ <1], neither were any significant interaction effects involving group (*p*'s > 0.26). The robust effect of cue and cue by block interaction supported the hypothesis that RTs are sensitive to task contingencies. The presence of learning was further supported by significant high vs. low reward (z-transformed) RT differences in all four groups on the second block (*t*'s > 2.71, *p*'s < 0.011: see Figure 1). Raw RT and error scores are presented in Table 2.

**Table 2:** Raw RT (ms units) and error scores (mean/standard deviation) for each group, for each block (1 and 2), and cue (high, mid, and low reward probability).

	**HC**	**MDD**	**BPD**	**BPE**
Block 1 Low RT	657.71 (115.85)	691.84 (155.07)	718.12 (186.63)	670.25 (158.89)
Block 1 Mid RT	657.40 (116.46)	700.02 (166.11)	739.03 (191.60)	683.16 (161.79)
Block 1 High RT	651.36 (117.03)	682.39 (157.19)	730.93 (189.76)	686.52 (177.71)
Block 2 Low RT	635.94 (112.85)	669.91 (153.75)	676.12 (159.03)	665.56 (165.75)
Block 2 Mid RT	625.67 (114.62)	659.35 (151.70)	665.75 (163.11)	655.66 (170.53)
Block 2 High RT	630.10 (121.57)	653.30 (152.25)	657.13 (159.34)	644.11 (156.42)
Block 1 Low Error	3.23 (3.33)	2.44 (2.11)	2.27 (2.18)	3.03 (2.83)
Block 1 Mid Error	2.91 (3.58)	2.68 (2.29)	2.20 (1.74)	2.97 (3.42)
Block 1 High Error	2.59 (2.60)	2.54 (2.45)	2.40 (1.98)	3.60 (3.87)
Block 2 Low Error	2.57 (2.94)	2.27 (2.06)	2.10 (1.79)	2.60 (2.67)
Block 2 Mid Error	2.39 (2.96)	1.66 (1.71)	2.07 (1.91)	2.34 (2.53)
Block 2 High Error	2.41 (3.21)	2.49 (2.42)	2.20 (2.21)	3.46 (3.56)

In terms of the number of points won overall, a significant main effect of block was observed [*F*_(1, 146)_ = 42.31, *p* < 0.001] which reflected a general increase in points scored in block 2, but no main effect of group [*F*_(3, 146)_ = 1.34, *p* = 0.26]. A marginal block by group interaction was also observed [*F*_(3, 146)_ = 2.68, *p* = 0.049]. Games-Howell non-parametric *post hoc* tests on the changing score from block 1 to block 2 revealed that healthy controls showed a relatively smaller increase in the number of points across blocks, and this was significantly lower than the BPD group (*p* = 0.014). No other significant findings were observed.

### Errors

**Figure 2:**
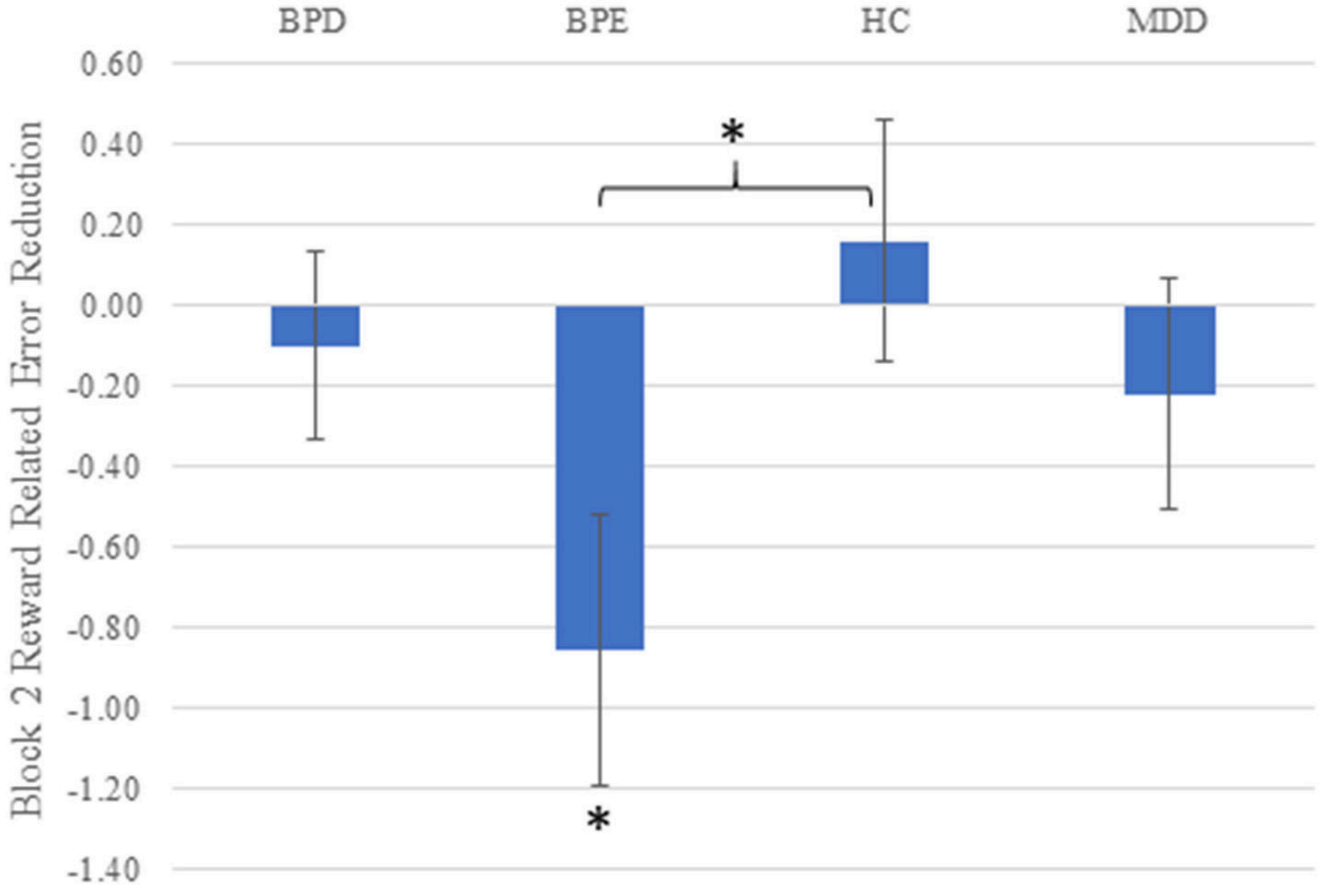
Reward-related error rate (high vs. low reward cue) on the second block in all four groups. Higher scores on the Y axis indicate more accurate responding for high vs. low reward cues. Error bars reflect standard error of the mean; asterisks reflect the mean value being significantly greater than zero (BPE), or the BPE vs. control difference being significant (*p*'s < 0.05).

Overall, all three groups showed similar rates of overall commission errors (χ^2^ < 1.2, *p*'s > 0.7 across both blocks and overall). Similar findings were seen with overall omission (“too late”) errors (*p*'s > 0.45). Following the RT analysis by focusing on relative errors for the high vs. low reward cue in the second block, Kruskal–Wallis tests revealed a main effect of group (χ^2^ = 9.26, df = 3, *p* = 0.026). The only *post-hoc* test reaching significance when corrected for the number of possible group (*n* = 6) comparisons was the difference between control and BPE individuals (*Z* = 2.76, *p* = 0.006), with BPE individuals making relatively more errors in the high reward than the low reward condition than the controls in the second block (see Figure 2). The BPE group also tended to make relatively more errors on high vs. low reward trials in the second block than the BPD group (*p* = 0.027) and the MDD group (*p* = 0.090). Looking at differential error rates within the groups, the BPE group was the only group to show a significant difference between the block 2 stimuli in terms of error rates, showing significantly higher error rates to the high compared to low reward stimuli (*Z* = 2.49, *p* = 0.013). The three other groups showed similar error rates on block 2 across stimuli. Finally, a difference between the groups on high vs. low error rate was not seen on the first block (χ^2^ = 3.77, df = 3, *p* = 0.29). We also investigated the impact of the number of prior manic episodes on the reward-related error rate within the BPE/BPD groups. A significant relationship was observed (ρ = −0.28, *p* = 0.027, *n* = 65), with relatively greater errors on the high reward trials being observed in patients who had experienced greater numbers of manic episodes.

### Relationship of speed and accuracy

**Figure 3:**
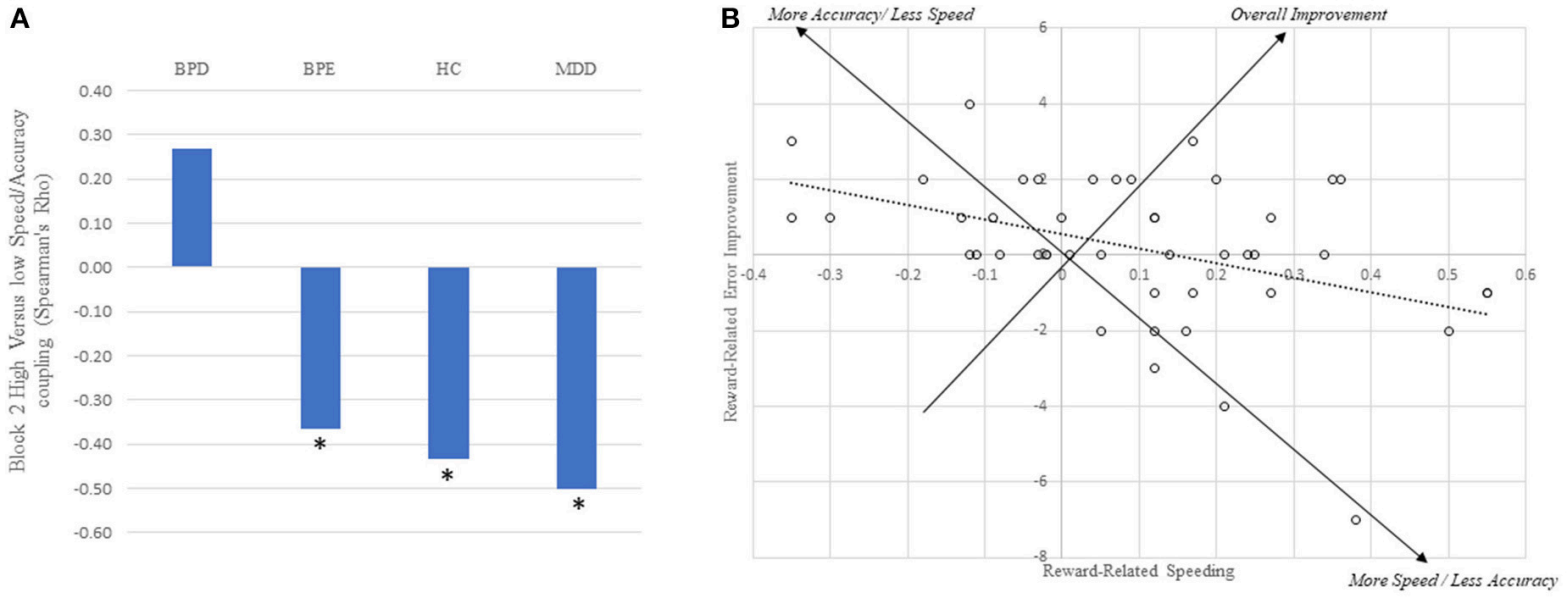
Trade-off between increases in speed and accuracy for the high vs. low cue. **(A)** Between subject relationship between reward-related speed and accuracy improvements across all four groups. Asterisks reflect correlations being significantly different from zero (*p'*s < 0.05). **(B)** Plot of the individual subject data for the healthy control group alone, revealing the trade-off between speed and accuracy improvements. Note that a large proportion of individuals are in the upper right-hand quadrant, suggesting overall reward-related improvements.

As described above, the strongest metric of reward-related speeding was high vs. low reward RT difference on the second block. If participants are differentially balancing speed and accuracy, we might expect that, across individuals, shorter RTs in the high reward condition would come at the cost of a relatively increased error rate. Across all individuals, this hypothesis was supported (ρ = 0.31, *p* < 0.001), and strongly in the HC (ρ = 0.43, *p* = 0.003) and MDD (ρ = 0.50, *p* = 0.001) individually. In the BPE group, the relationship was significant if numerically smaller (ρ = 0.37, *p* = 0.031), and was not present in the BPD group (ρ = −0.27, *p* = 0.15: see Figure 3).

To model this finding more formally, we constructed an ordinal logistic regression model in which we aimed to predict the (high-low, block 2) errors from the amount of speeding differentially across the groups. The model confirmed a large main effect of high vs. low RT speeding (Wald = 13.00, *p* < 0.001). In addition, BPE showed main effect (Wald = 5.50, *p* = 0.019) (i.e., overall difference in high vs. low errors), but the BPE^*^RT speeding interaction term was not significant (*p* = 0.35). By contrast, the BPD group showed no significant main effect (*p* = 0.29) but significant interaction term (Wald = 9.089, *p* = 0.003).

### General linear model

The Q value of the cue, as derived from the RL model, was related to RT as evidenced by a beta statistic that was significantly different from zero across all individuals [*t*_(149)_ = 6.32, *p* < 0.001]: trials where the cue value was higher had shorter RTs. Value-related RT speeding was correlated with block 2 high vs. low RT difference across all participants (r = −0.49, *p* < 0.001). No main effect of group was seen on this variable [*F*_(3, 146)_ = 1.68, *p* = 0.17]. Moreover, a Bayesian test of null hypothesis of currently depressed individuals (i.e., all MDD/BPD participants) vs. currently non-depressed individuals (all HC/BPE participants) revealed strong evidence for the null hypothesis of no difference between the groups (Bayes Factor = 12.91). Finally, of the three significant findings in the ordinal logistic regression model described in section Relationship of Speed and Accuracy, the main effect of value-related speeding (Wald = 4.91, *p* = 0.027), main effect of BPE (Wald = 3.41, *p* = 0.065) and BDE*value-related speeding (Wald = 5.88, *p* = 0.015), all at least trended in the same direction as previously, while no further significant findings were observed.

### Medication effects

Including the presence of antipsychotic medications or mood stabilizers in the ordinal logistic regression model described in section General Linear Model reduced the effect of the BPE main effect (*p* = 0.079 or *p* = 0.2 respectively), while mood stabilizers themselves had a trend level effect (*p* = 0.063). A clearer influence was seen with overall medication load, which itself had a significant main effect (Wald = 4.23, *p* = 0.040) and load by speeding interaction (Wald = 4.58, *p* = 0.032). In this model, the BPE main effect was also reduced to marginal (*p* = 0.058). In all of these models, the significance of the BPD by speeding interaction was unaffected. Importantly, when the effect of antipsychotic medication, mood stabilizers, or medication load were examined in the BPE group alone, no significant differences in relative error rate were observed (all *p*'s>0.47). In other words, differential medication status within BPE participants was associated with similar patterns of reward-related error rates.

## Discussion

In the present study, we evaluated four groups of participants - euthymic and depressed patients with bipolar disorder, unipolar depressed individuals and healthy controls—on a motivated reaction time paradigm. Across all individuals, significant, and similar RT speeding was observed across all four groups. An analysis using RT differences was confirmed with simple reinforcement learning (RL) model instantiated within a GLM. Second, different pattern of cue-related errors was observed in the BPE group: BPE individuals showed higher rates of errors in the high vs. low reward condition on block 2, whereas no other group showed this effect. Complementing this finding, greater numbers of manic episodes in the bipolar groups also predicted higher error rates in the high vs. low reward condition, suggesting that there may be dimensions of illness severity or chronicity which may generalize across the bipolar groups.

Accounting for differences in RT clarified the relative error rate finding further, as there were substantial individual differences in RT/errors. Controls and MDD groups showed strong negative relationships between high vs. low error difference scores, and high vs. low RT difference scores. This suggests that individuals adopt different strategies: on one end of the spectrum, they might reduce RTs dramatically for high reward cues at the cost of increased errors; at the other end, they might reduce RTs slightly and show relatively low error rates following the high reward cue. By contrast, both of the BD groups showed a complication of this predicted relationship: the BPE group showed an overall increase in errors in the high vs. low reward cue, while the BPD group showed a decoupling of high vs. low reward error rate and high vs. low RT.

### Comparison with previous findings

Similar findings to the present were obtained by Mueller et al. (31): youth with pediatric bipolar disorder showed a higher error rate under the incentive condition of an anti-saccade task than healthy controls, while similar performance between the groups was seen on the no incentive condition, and on a pro-saccade task. This type of finding is compatible with our observations, insofar as the task incentives were associated with impaired performance in the bipolar group.

Existing data on the speed/accuracy trade off (SAT) in major depression are somewhat complex. Although MDD patients might be expected to favor accuracy over speed, Dillon et al. (32) demonstrated, using drift diffusion modeling of a flanker task, that a slower executive control process is offset by a slower prepotent bias. As a result, the SAT seen in MDD patients was roughly similar to that seen in controls. We were unable to replicate the finding of *enhanced* performance in MDD patients that we had previously reported (14). The present study cannot be considered an exact replication attempt, because the age of the participants was different between the samples (participants in the present sample were around ~10 years younger), and different medications had been prescribed. Age may be a relevant dimension for further investigation because reanalysis of our previous findings suggested that age was positively related to overall errors and reaction time variability in the healthy control group (*r*'s = 0.44–0.50) but not in the patient group (*r*'s = −0.08: unpublished data). As the age range of the present sample is only partially overlapping with the previous sample, it is difficult to perform a direct replication of this finding here. Overall, a unifying conclusion of both datasets, which would be directly testable, is that MDD and/or antidepressant medications may prevent decline in overall RT performance in individuals >45 years old.

Nevertheless, the present findings strengthen an important conclusion of the previous study: namely, that reward-predictive cues can exert similar speeding effects on motivated behavior in depressed and healthy individuals. The present study was adequately powered to detect effects of similar magnitude to those reported in previous studies of psychopathology with this task [d = 0.77–1.29: (13, 14)]. These findings contribute to a growing literature describing areas of intact reward processing in MDD (33), and provide contrast with paradigms which appear to be more reliably sensitive (34, 35). Although details of the experimental design within the reinforcement learning framework may be relevant to understanding these discrepancies, it may also be that theoretical accounts of motivation that extend beyond a focus on the arrangement of stimuli, responses requirements and reinforcement contingencies may be insightful in determining why some paradigms are sensitive to differences related to depression and some are not (36).

### Neurocomputational basis

If the spirit of the impulsivity construct is one of sub-optimal decision making, then considering impulsivity in terms of the SAT might emphasize a failure to optimize RT in order to maximize utility, rather than simple premature or hasty responding. But whether or not healthy individuals show an optimal trade-off of speed and accuracy is also debated (37), and can be considered in terms of the drift diffusion model. This provides a framework to allow a derivation of optimal performance on decision paradigms across different levels of sensorimotor signal to noise ratio [SNR—(37)]. In addition, Manohar et al. (38) demonstrated that reward can provide a general improvement in performance rather than biasing toward speed or accuracy. In our data, there was a strong negative correlation between the degree of RT speeding and enhanced error rates. For a given SNR, this might be explained by proposing that individuals differ considerably regarding the relative utility of reward and punishments available on the task. For the HC and MDD groups, the pattern of findings follows what might be expected if participants showed some variation in how the response threshold parameter is set (i.e., more or less liberally) in order to promote reward rate or reduce punishment rate. It would also be necessary to propose an increase in SNR in the high reward condition [see (38)], as performance was generally better (faster and as accurate) here than in the low reward conditions. By contrast, the bipolar participants did not conform to this pattern. Both bipolar groups shortened their RTs overall in the high reward condition, suggesting a loosening of the decision threshold, but individual differences in this shortening were not always accompanied by predictable changes in error rate. It may be that some bipolar participants showed a decrease in SNR during the high reward condition. In combination with looser decision thresholds, a decrease in SNR might lead to increased error rates and shorter reaction times—the pattern seen in the BPE group. A more subtle effect of reducing SNR in the high condition might simply be to reduce the coupling between error rates and RT, which was the finding seen in the BPD group. In other words, the same type of explanation (i.e., a relative decrease in SNR in high reward conditions) might account for both the observed main effect (BPE) or the group by speeding interaction (BPD). High reward cues in the bipolar groups may affect behavior by a number of different mechanisms, including reduced selective attention, or the engagement of competing but irrelevant responses. Either possibility may appear to decrease SNR in the high reward condition.

Clearly then, our data do not support the idea that there is a simple change in response threshold in mood disorders, but previous studies which manipulated serotoninergic neurotransmission using acute tryptophan depletion (ATD) can be largely explained in this way (12, 26). Specifically, ATD was associated with a reduction in reward-related speeding, particularly in highly impulsive individuals (12) and individuals carrying the ss allele of the 5-HT transporter gene (26). In both of these studies, errors broadly tended to follow what would be expected by RT speeding: for example, ATD was associated with overall reduced error rates in ss individuals (26), and fewer errors on high compared to low reward conditions (12).

### Limitations

Medication was a confound in the present study, and the presence of psychiatric control groups (e.g., MDD) did not allow us to correct for this completely: the effect seen in the BPE group (increased errors in the high vs. low condition) did not survive correction for medication load, although the effect seen in the BPD group (error by RT interaction) did. However, as the BPE group showed similar error rates if medicated by antipsychotics, mood stabilizers or higher overall doses than if they were not medicated in this way, it seems most likely that the reduction in significance relates to confounding and a consequent loss of statistical power rather than a particular effect of medication on performance. Finally, the findings of the present study do not support the contention that antidepressants improve performance which was an interpretation of our previous work (14), but it remains possible that the effect of anti-depressants may be age dependent, as described above.

One limitation of the paradigm is that it is broadly reward-focused. Recent studies have manipulated reward and punishment independently within the context of compatible motivated RT paradigms (27, 39). Future studies could usefully examine the generalization of these finding across different experimental contingencies. One straightforward manipulation would be to add another block (or more) of trials: this would allow more precise measurement of asymptotic performance, particularly of error rates which are low and thus may be difficult to estimate accurately. Finally, future studies could also explore drift diffusion modeling to verify some of our conclusions, although some more substantial alterations to the experimental design may be necessary to constrain the number of potential free parameters (40).

### Summary

In summary, we emphasize two primary contributions of the present work. First, the findings confirm the presence of reward-related speeding within mood disorders: this finding is in line with our previous work, but does not support the hypothesis of motivational impairment within depressed individuals. Second, we provide evidence for an alteration in a reward-related trade-off between speed and accuracy within individuals with BD. Our favored interpretation of this finding is that individuals with BD may show a decrease in sensorimotor SNR under high reward expectation, as opposed to individuals with MDD and HC who show increased SNR. Together, the findings suggest a novel avenue for research into impulsivity in mood disorders.

## Author contributions

HC was involved in the framing of the study, data analysis, and write up of the manuscript. JF was involved in data analysis and editing of the manuscript. HA and RS was involved in data collection and management. JA was involved in study design, organization, and data collection. BS was involved in study design and editing of the manuscript. MP was involved in study design, organization, framing of the study, and write up of the manuscript.

### Conflict of interest statement

The authors declare that the research was conducted in the absence of any commercial or financial relationships that could be construed as a potential conflict of interest.
