# Supplementary material for: Haste or Speed? Alterations in the Impact of Incentive Cues on Task Performance in Remitted and Depressed Patients With Bipolar Disorder
Source: Front Psychiatry. 2018 Sep 3;9:396. doi: 10.3389/fpsyt.2018.00396 (PMC6129608; doi:10.3389/fpsyt.2018.00396)
Supplement: Supplementary file 1 [file Table_1.docx]

|  | MDD | BPD | BPE |
| --- | --- | --- | --- |
| ALPRAZOLAM | 1 | 2 | 0 |
| ARIPIPRAZOLE | 1 | 4 | 7 |
| ATOMOXETINE | 1 | 1 | 1 |
| BENZTROPINE | 0 | 2 | 2 |
| BUPROPION | 6 | 4 | 3 |
| BUSPIRONE | 0 | 1 | 1 |
| CARBAMAZEPINE | 0 | 3 | 2 |
| CHLORPROMAZINE | 1 | 0 | 0 |
| CITALOPRAM | 6 | 2 | 3 |
| CLOMIPRAMINE | 0 | 1 | 0 |
| CLONAZEPAM | 7 | 3 | 1 |
| CLORAZEPIC ACID | 0 | 1 | 0 |
| CLOZAPINE | 0 | 0 | 1 |
| DESIPRAMINE | 1 | 0 | 0 |
| DOXEPIN | 0 | 1 | 0 |
| DULOXETINE | 2 | 1 | 1 |
| ESCITALOPRAM | 4 | 0 | 1 |
| FLUOXETINE | 5 | 1 | 2 |
| FLUVOXAMINE | 1 | 0 | 0 |
| GABAPENTIN | 3 | 1 | 0 |
| HALOPERIDOL | 0 | 1 | 1 |
| HYDROXYZINE | 3 | 0 | 0 |
| LAMOTRIGINE | 2 | 10 | 7 |
| LISDEXAMFETAMINE | 0 | 0 | 1 |
| LITHIUM | 1 | 5 | 13 |
| LORAZEPAM | 2 | 2 | 2 |
| METHYLPHENIDATE | 2 | 0 | 0 |
| MIRTAZAPINE | 3 | 0 | 0 |
| OLANZAPINE | 0 | 3 | 1 |
| OXCARBAZEPINE | 0 | 0 | 2 |
| PAROXETINE | 1 | 0 | 0 |
| PERPHENAZINE | 0 | 2 | 0 |
| PREGABALIN | 1 | 0 | 0 |
| QUETIAPINE | 0 | 4 | 9 |
| RISPERIDONE | 1 | 3 | 1 |
| SELEGILINE | 1 | 0 | 0 |
| SERTRALINE | 2 | 2 | 1 |
| TEMAZEPAM | 1 | 1 | 0 |
| TOPIRAMATE | 0 | 1 | 1 |
| TRAZODONE | 6 | 1 | 4 |
| VALPROIC ACID | 0 | 3 | 2 |
| VENLAFAXINE | 3 | 3 | 0 |
| ZIPRASIDONE | 0 | 5 | 3 |

Table S1: List of medications taken for each of the three patient groups. Numbers refer to the numbers of participants medicated with each medication: some participants were taking >1 medication.
